# Psychometric properties of palliative care outcome measures: a multi-centre study

**DOI:** 10.1186/s41687-025-00954-6

**Published:** 2025-10-16

**Authors:** Animut Alebel Ayalew, Sabina Clapham, Katherine Clark, Farina Hodiamont, Lisa Redwood, David Currow

**Affiliations:** 1 Palliative Care Outcomes Collaboration, Australasian Health Outcomes Consortium, Faculty of Science, Medicine and Health, University of Wollongong, Wollongong, NSW Australia; 2 Northern Sydney Local Health District Supportive and Palliative Care Network, Royal North Shore Hospital, Sydney, NSW Australia; 3 Northern Clinical School, The University of Sydney, Sydney, NSW Australia; 4 Department of Palliative Medicine, LMU University Hospital, LMU Munich, Munich, Germany; 5 College of Medicine and Public Health, Flinders University, Bedford Park, South Australia, Australia; 6 University of Wollongong, Innovation Campus, Innovation Wy, North Wollongong, NSW 2500 Australia

**Keywords:** IPOS, PCPSS, Reliability, SAS, Validity

## Abstract

**Background:**

Periodic evaluation of the psychometric properties of palliative care outcome measures is essential to ensure accurate assessment of patient outcomes and to support ongoing improvements in care quality. This study aimed to assess the psychometric properties of three tools: the Integrated Palliative Care Outcome Scale (IPOS), the Symptom Assessment Scale (SAS), and the Palliative Care Problem Severity Score (PCPSS).

**Methods:**

We conducted a multicentre study using de-identified data collected from 378 participants. Internal consistency was assessed using Cronbach’s alpha for all tools, and confirmatory factor analysis was performed for the IPOS. Convergence and discriminant validity were examined for the SAS and PCPSS by analysing the correlations between similar and dissimilar items, respectively. Lastly, known-groups comparison validity was assessed.

**Results:**

Of the 378 participants, 54.5% were male, and most (77.5%) had cancer. Internal consistency was good for the IPOS total (α = 0.81), acceptable for the SAS (α = 0.70), and marginal for the PCPSS (α = 0.65). A strong correlation was observed between the pain construct of SAS and PCPSS (*r* = 0.74). Both the PCPSS and SAS effectively discriminated symptoms between palliative care phases and settings of care in known-group comparisons. The physical domain of the IPOS demonstrated good discriminative ability in differentiating symptoms between cancer and non-cancer patients. Our data confirmed the three theoretical domains of the IPOS—physical, emotional, and informational.

**Conclusion:**

The IPOS demonstrated higher reliability using internal consistency, whereas SAS and PCPSS showed good validity in known group comparisons. These findings may inform the selection of context-appropriate palliative care outcome measures.

## Background

Patient outcome measurement is crucial for enhancing the quality of care and demonstrating the value of palliative care within health systems [1]. In recognition of this, the 2016 White Paper on outcome measurement strongly recommended the implementation of tools with sound psychometric properties [1]. Australia has a national palliative care program known as the Palliative Care Outcomes Collaboration (PCOC), which is an outcome and benchmarking program that embeds point-of-care patient- (and, at times, proxy-) reported outcomes. PCOC uses five clinical assessment tools: the PCOC Symptom Assessment Scale (PCOC-SAS) [2, 3], the Palliative Care Problem Severity Score (PCPSS) [4], the Palliative Care Phase [5], the Australia-modified Karnofsky Performance Status (AKPS) [6], and the Resource Utilisation Groups-Activities of Daily Living (RUG-ADL) [7]. These tools enable the PCOC to summarise individual needs and benchmark patient and service outcomes, thereby facilitating nationwide quality improvement in palliative care.

Among internationally recognised palliative care outcome measures, the Integrated Palliative Care Outcome Scale (IPOS) is one of the most widely used tools globally for patient-reported outcomes (PROs) [8]. Although IPOS is not routinely used by the Australian PCOC for national benchmarking, it has been periodically used within Australia to assess patients’ experience of pain and other symptoms [9]. Unlike the PCOC-SAS and PCPSS, which primarily focus on physical symptoms, IPOS is a multi-dimensional tool that addresses emotional symptoms, practical issues, and information needs.

Regarding administration, both IPOS and PCOC-SAS can be rated by patients or proxies (clinicians or families), whereas the PCPSS is a clinician-rated tool. It is widely accepted that patient-reported measures are considered the gold standard. IPOS has demonstrated good reliability and validity [10–15]. A previous Australian study reported that the older version of IPOS, known as the Palliative Care Outcome Scale (POS), had very good validity [16]. PCOC-SAS and PCPSS have also been previously evaluated and confirmed as reliable and valid tools in Australia [2, 4, 17].

While prior studies have reported the psychometric properties of each tool separately, none has provided a detailed comparison of these tools in a single study [2, 4, 6, 11, 15, 17, 18]. In addition, despite the routine use of SAS and PCPSS in more than 200 Australian palliative care services, the psychometric properties of these tools have never been thoroughly evaluated. Additionally, neither SAS nor PCPSS has previously been compared with the IPOS. This study aims to address this evidence gap by examining the psychometric properties of the tools routinely used for benchmarking by the Australian PCOC (PCOC-SAS and PCPSS) in comparison with IPOS, as one of the most widely used and well-accepted international measures. To our knowledge, this is the first study designed to assess the psychometric properties of these widely used interrelated tools.

## Methods

### Study setting and data sources

**Fig. 1:**
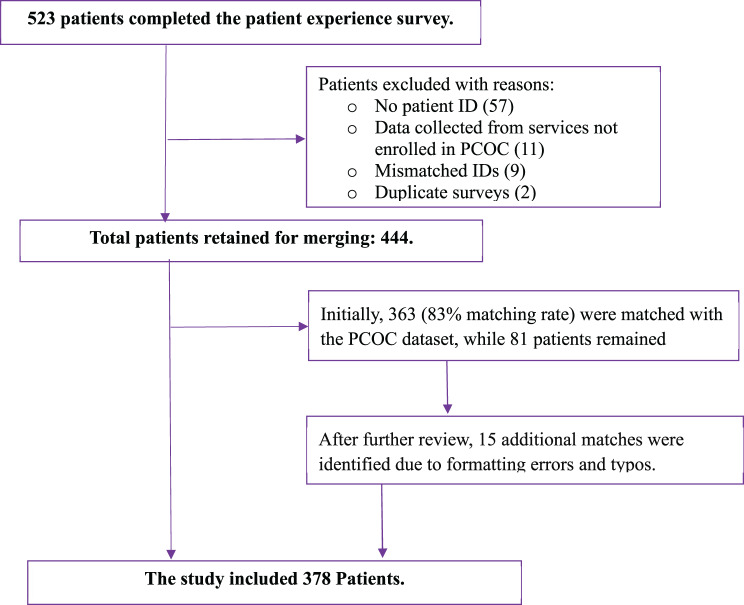
Flowchart of data merging process to evaluate the psychometric properties of SAS, PCPSS, and IPOS

This study used two datasets. The first dataset was obtained from a survey conducted by PCOC to assess patients’ experiences with palliative care, collected between October 2020 and February 2021. The second dataset was obtained from the Australian PCOC data, collected between July and December 2020, and included a consecutive cohort of data collected as part of routine clinical care. These two datasets were then merged for analysis (Fig. 1).

PCOC is a voluntary national quality improvement initiative funded by the Australian Government Department of Health, Disability, and Ageing. Palliative care services across Australia participate in this quality improvement and benchmarking program. The PCOC longitudinal dataset is organised into three linked data levels: patients, episodes, and phases. Patient data include demographics that are usually recorded at enrolment and remain unchanged, except for postal codes due to address changes. Episode data relate to the period a patient spends within a single institution, as defined by PCOC. Phase data involve an ongoing clinical assessment of the person.

### Population and data merging

All adults (age ≥18 years) who participated in the patient experience survey conducted between October 2020 and February 2021 were eligible for inclusion. The data merging process was as follows: Initially, all patients who participated in the patient experience survey were identified (dataset 1). Secondly, the demographics, SAS scores, and PCPSS scores for these participants (dataset 1) were extracted from routinely collected PCOC data (dataset 2) using their unique identification (ID). Third, the two datasets were merged using ID (Fig. 1). Demographic, SAS, and PCPSS data were collected concurrently (data 2), whereas IPOS scores were collected at a different time. The inconsistences were addressed during statistical analysis.

### Sample size adequacy

We included all individuals with complete information in the dataset but estimated required sample size using an online calculator (wnarifin.github.io) to ensure adequacy. Assumptions for this estimation included a minimum acceptable Cronbach’s alpha (H_0_) of 0.7, an expected Cronbach’s alpha (H_1_) of 0.62, which was taken from a previous Australian study assessing the reliability of the SAS [2], a significance level (α) of 0.05, a power (1-β) of 80%, seven items (k) for the PCOC SAS, and an anticipated non-response of 10%. The initial calculated sample size was 330; however, accounting for 10% non-response, the final sample size was adjusted to 367. The number of included patients (*n* = 378) exceeded the requirement, confirming adequacy.

### Study variables and measurements

The study variables were broadly categorised into demographic and clinical variables. Demographic variables included age, sex, country of birth, preferred language, and setting. Clinical variables consisted of diagnosis, palliative care phase type, PCOC SAS item scores, PCPSS item scores, IPOS item scores, and AKPS scores.

### Instruments

- **PCOC SAS** is an 11-point scale, completed by either patients or their proxies and used to assess patient distress from seven physical symptoms, with responses for each symptom ranging from zero (absent) to 10 (worst possible). The symptoms include pain, sleeping difficulty, appetite problems, nausea, bowel problems, breathing problems, and fatigue. The final distress levels for each symptom were classified into four categories: absent (zero), mild (one to three), moderate (four to seven), and severe (eight to ten) [2].
- **PCPSS** is a clinician-rated scale that assesses the severity of problems using four items: pain, other symptoms, psychological/spiritual problems, and family/carer problems, rated on a 4-point categorical scale (absent, mild, moderate, severe) [4].
- **IPOS** is a 17-item scale across three domains: physical symptoms (10 items), emotional symptoms (4 items), and communication and practical issues (3 items). The physical symptoms domain includes pain, shortness of breath, weakness, vomiting, poor appetite, constipation, soreness of the mouth, and poor mobility. Furthermore, the emotional symptom domain assessed patient anxiety, family anxiety, patient depression, and feelings of peace. The third domain is about communication and practical problems, and it assesses issues related to being able to share feelings, information access, and practical problems. In this study, we used the patient version of IPOS, which is available at http://www.pos-pal.org.
- **AKPS** is an ordinal scale that classifies functional status into 11 categorical levels, ranging from 0 (death) to 100 (normal). These levels are based on the patient’s ability to engage in activities, work, and self-care [6].
- **The Palliative Care Phase** classifies the care needs of patients and their families/carers. Patients are classified into four phases: stable, unstable, deteriorating, and terminal [5].

### Missing data management

No data were imputed, and a complete case analysis was considered since the missing values for almost all variables were less than 5% (rule of thumb).

### Statistical analysis

The demographic and clinical characteristics of patients were summarised using frequencies and percentages for categorical variables and means, or medians, for continuous variables. Stacked bar charts and a correlation plot were used for data visualisation. The psychometric properties of the three tools were evaluated using different methods, including reliability and validity, as detailed below.

### Reliability

was evaluated using Cronbach’s alpha coefficient. Results were interpreted as excellent (α ≥ 0.9), good (α = 0.8–0.9), acceptable (α = 0.7–0.8), marginal (α = 0.6–0.7), poor (α = 0.5–0.6), or unacceptable (α < 0.5) [19].

### Structural validity

A confirmatory factor analysis (CFA) was conducted using one-factor, two-factor, and three-factor solutions based on predetermined sub-scales of IPOS [20]. The goodness of fit for the CFA model was assessed using chi-square test statistics, root-mean-squared error of approximation (RMSEA), Tucker-Lewis Index (TLI), and the Comparative Fit Index (CFI). A CFI > 0.9 and RMSEA < 0.08 were considered indicators of an acceptable model [21].

### Construct validity

Convergent and discriminant validity were performed only for SAS and PCPSS, as the data collection period for IPOS and these two tools differed slightly, and a direct comparison could result in biased estimation. Convergent validity was evaluated by examining the correlation between similar items (i.e., SAS pain and PCPSS pain). We hypothesised moderate to strong positive correlations would exist between similar items. Conversely, the discriminant validity was examined by checking the correlations between different items. Weak correlations were hypothesised between different items, and results were interpreted based on the following classifications: *r* < 0.2 (very weak), 0.2 ≤ *r <* 0.4 (weak), 0.4 ≤ *r* < 0.6 (moderate), 0.6 ≤ *r <* 0.8 (strong), and *r* ≥0.8 (very strong) [22].

Known group comparisons were used to measure the instrument’s ability to distinguish among distinct groups. Each scale item was compared across selected categorical variables, including phase type (stable vs unstable/deteriorating/terminal), primary diagnosis (cancer vs non-cancer), and care settings (inpatient vs community). It was hypothesised that significant differences would be observed among unstable/deteriorating/terminal phases compared to the stable phase; in cancer patients compared to non-cancer patients; and inpatient setting compared to community setting. Special consideration was taken in the IPOS known group validity by excluding participants (*n* = 53) whose episode started before October 2020; the goal is to align with the patient experience survey date and has been mentioned in Table 5 footnote.

The total and physical scores of IPOS satisfied the normality assumption as shown by the Shapiro-Wilk test, and the independent t-test was employed for known-group comparisons. The Mann-Whitney U test was used for known-group comparisons of other scores, as the normality assumption was violated according to the Shapiro-Wilk test. The sample size suitability for factor analysis was evaluated using the Kaiser-Meyer-Olkin test. The weighted least squares mean and variance-adjusted estimation method instead of maximum likelihood (ML) was employed for the final CFA model because our data was ordinal. All statistical analyses were conducted using Stata version 18 and R version 4.4.0.

## Results

### Demographic and clinical characteristics of participants

The study included 378 patients receiving palliative care in Australia, of whom 54.5% (*n* = 206) were male. Most (72.2%; *n* = 273) received palliative care in inpatient settings, and 75.9% (*n* = 287) were born in Australia and used English as their first language (96.8%; *n* = 366). Three quarters of the cohort (77.5%; *n* = 293) had cancer as their life-limiting illness. A significant age distribution was concentrated in the 70–79 age range (33.9%; *n* = 128), with the AKPS scores indicating that a considerable proportion of patients (43.1%; *n* = 163) required significant assistance. Forty percent of patients were in the stable phase during the initiation of palliative care (Table 1).

**Table 1:** Demographic and clinical characteristics of study participants to compare the psychometric properties of IPOS, SAS, and PCPSS

Variable	Frequency (N)	Percentage (%)
**Setting**		
Inpatient	273	72.2
Community	105	27.8
**Sex**		
Male	206	54.5
Female	171	45.2
Missing	1	0.3
**Country of birth**		
Australia	287	75.9
Overseas	90	23.8
Missing	1	0.3
**Preferred language**		
English	366	96.8
Non-English	6	1.6
Missing	6	1.6
**Diagnosis**		
Cancer	293	77.5
Non-cancer	84	22.2
Missing	1	0.3
**Age group in years**		
0–49	22	5.8
50–59	26	6.9
60–69	72	19.0
70–79	128	33.9
80–89	104	27.5
90+	25	6.6
Missing	1	0.3
**AKPS at baseline**		
10–40	163	43.1
50–60	174	46.0
70–100	33	8.7
Missing	8	2.1
**Palliative care phase at baseline**		
Stable	154	40.7
Unstable	59	15.6
Deteriorating	125	33.1
Terminal	38	10.1
Missing	2	0.5

### Symptom distress and problem severity scores

**Fig. 2:**
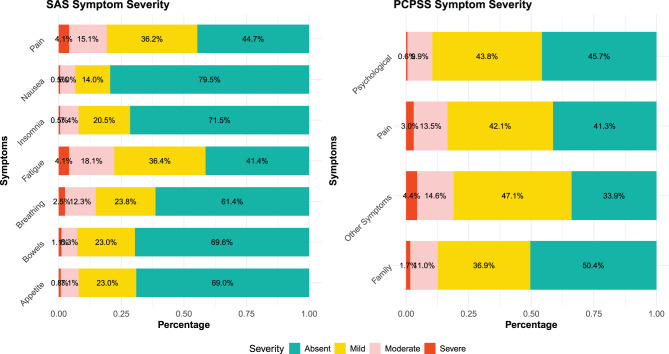
Distribution of symptom distress and problem severity levels assessed using the SAS and the palliative care problem severity score

More than half (54.3%) of the patients were assessed by health professionals as having experienced mild to severe psychological or spiritual issues, and 58.7% of the patients experienced mild to severe pain symptoms according to the PCPSS. Half of the families or carers (49.6%) experienced mild to severe problems related to patient conditions or needs. When the SAS scores were examined, more than half of the patients (55.3%) experienced mild to severe distress from pain. Mild to severe distress due to sleeping difficulties (insomnia) was present in 28.9% of patients. More than two-thirds of the patients did not report distress secondary to bowel (69.6%) or appetite (69.0%) related issues (Fig. 2).

### The structural validity of IPOS

**Fig. 3:**
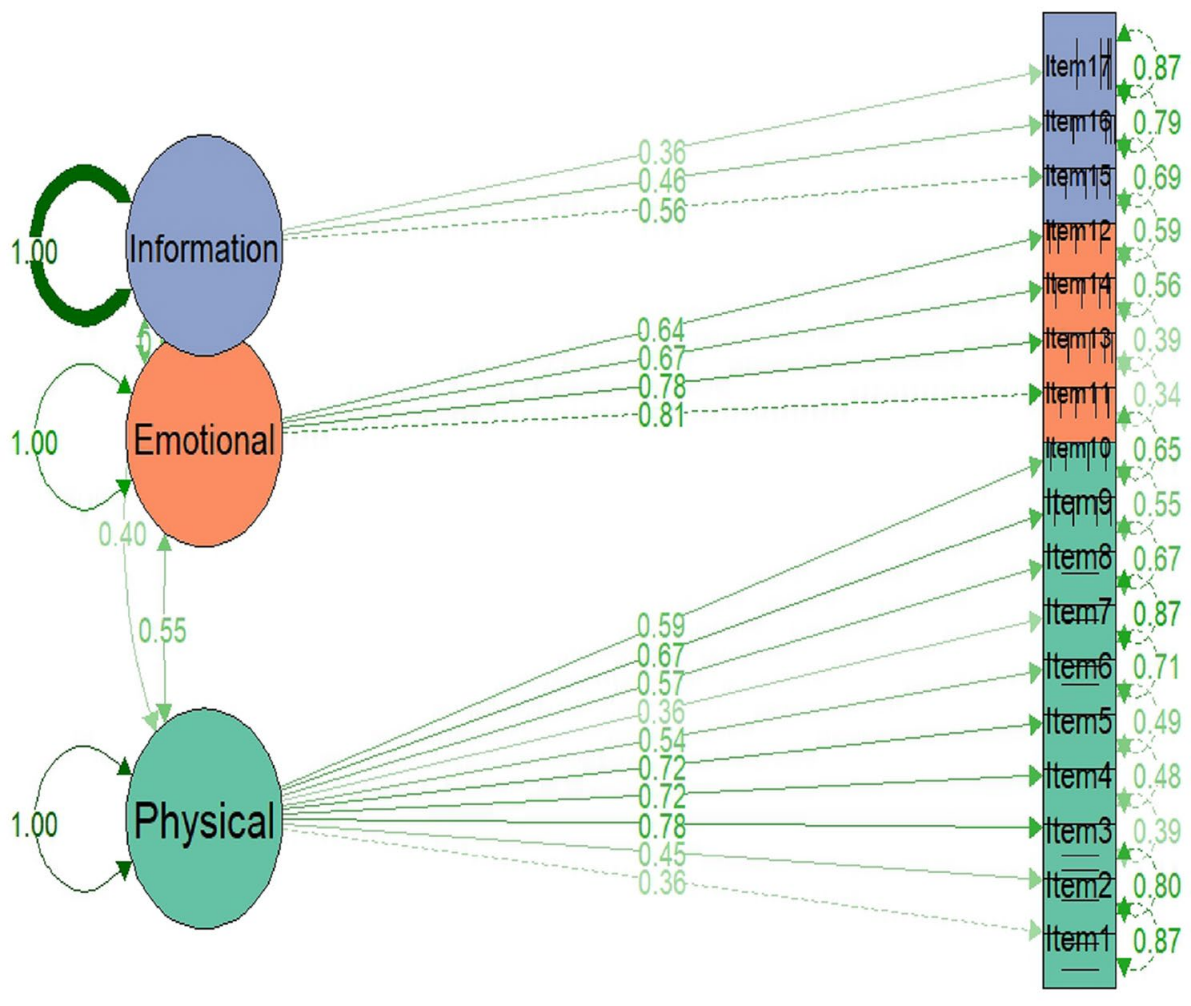
A path diagram of a three-factor model in confirmatory factor analysis for physical, emotional, and information and practical issues of IPOS

The prevalence of symptoms for each IPOS item ranged from 23.2% for vomiting to 90.9% for family anxiety among patients who reported a score of ‘slightly’ (1) or higher (Table 2). The one-factor, two-factor, and three-factor models were fitted (Table 3). The three-factor model showed lower chi-Square values and RMSEA, along with higher CFI (CFI = 0.94) and TLI (TLI = 0.931) values. While the CFI and TLI values of the three-factor model almost achieved the standard of a good fit, the chi-Square remained significant, which is expected with larger sample sizes. The three-factor CFA indicates that factor loadings for the physical domain ranged from 0.36 to 0.78, for the emotional domain from 0.64 to 0.81, and for the information and practical issues domain from 0.36 to 0.56. Accordingly, good construct validity was observed in the physical and emotional domains of the tool (Fig. 3).

**Table 2:** Descriptive presentation of integrated palliative care outcome scale (IPOS)

	Proportion of (%) response for each value score					Missing (%)	Prevalence (≥1)
Items	Not at all (0)	Slightly (1)	Moderately (2)	Severely (3)	Overwhelmingly (4)		
**Physical symptoms**							
Pain	18.8	25.5	31.3	18.3	6.1	4.5	81.2
Shortness of breath	32.2	21.6	28.1	12.3	5.7	3.2	67.8
Weakness	7.4	13.7	32.1	35.1	11.8	3.4	92.6
Nausea	54.7	19.5	15.9	6.9	3.0	3.7	45.3
Vomiting	76.8	13.4	7.3	2.0	0.6	5.3	23.2
Poor appetite	31.6	18.1	27.2	17.9	5.2	3.7	68.4
Constipation	38.7	22.0	24.2	10.6	4.5	5.0	61.3
Dry mouth	31.6	21.7	29.4	12.9	4.4	3.7	68.4
Drowsiness	15.1	22.2	35.6	19.7	7.4	3.4	84.9
Poor mobility	10.5	20.2	30.1	26.0	13.3	4.2	89.5
**Emotional symptoms**							
Patient anxiety	23.6	21.2	27.5	16.8	11.0	3.7	76.4
Family anxiety	9.1	11.9	24.6	32.3	22.1	4.2	90.9
Patient depression	34.9	26.4	22.0	10.7	6.0	3.7	65.1
Feeling at peace	15.4	37.1	23.4	14.6	9.6	3.7	84.6
**Communication/practical issues**							
Sharing feelings	30.3	29.0	16.7	15.8	8.2	3.2	74.2
Information	40.8	44.0	8.4	4.1	2.7	2.6	64.1
Practical problems	47.9	32.1	10.3	3.2	6.6	7.7	57.3

*Footnote:* The prevalence of symptoms for each IPOS item was calculated among patients reporting a score of ‘slightly’ (1) or higher

**Table 3:** Results of the confirmatory analysis of IPOS

Index of fit	One-factor	Two-factor	Three-factor
Number of items	17	17	17
Chi-Square	606.722	362.785	350.354
χ^2^-df	119	118	116
χ^2^ -P-value	0.000	0.000	0.000
CFI	0.877	0.938	0.941
TLI	0.860	0.929	0.931
RMSEA	0.118 (0.109, 0.128)	0.084 (0.074, 0.094)	0.083 (0.073, 0.093)

### Internal consistency and inter-item correlation

Cronbach’s alpha for the IPOS was calculated for the three sub-domains as well as for the entire tool. The Cronbach’s alpha value for the physical domain was 0.76 and for emotional domain 0.77, while the value for communication and practical issues was 0.35. The overall Cronbach alpha for the entire IPOS tool was 0.81. The Cronbach’s alpha for the PCOC SAS was 0.70, and for the PCPSS was 0.65 (Table 4).

**Table 4:** Internal consistency and inter-item correlation for IPOS, SAS, and PCPSS

Tools	Number of items	Cronbach’s alpha (α)	Inter-item correlation lowest and highest
IPOS			
Physical domain	10	0.76	0.10, 0.64
Emotional domain	4	0.77	0.25, 0.57
Communication and practical issues	3	0.35	0.11, 0.26
IPOS total	17	0.81	−0.10 to 0.64
PCOC SAS	7	0.70	0.08, 0.43
PCPSS	4	0.65	0.15, 0.48

### Convergent and discriminant validity between SAS and PCPSS

**Fig. 4:**
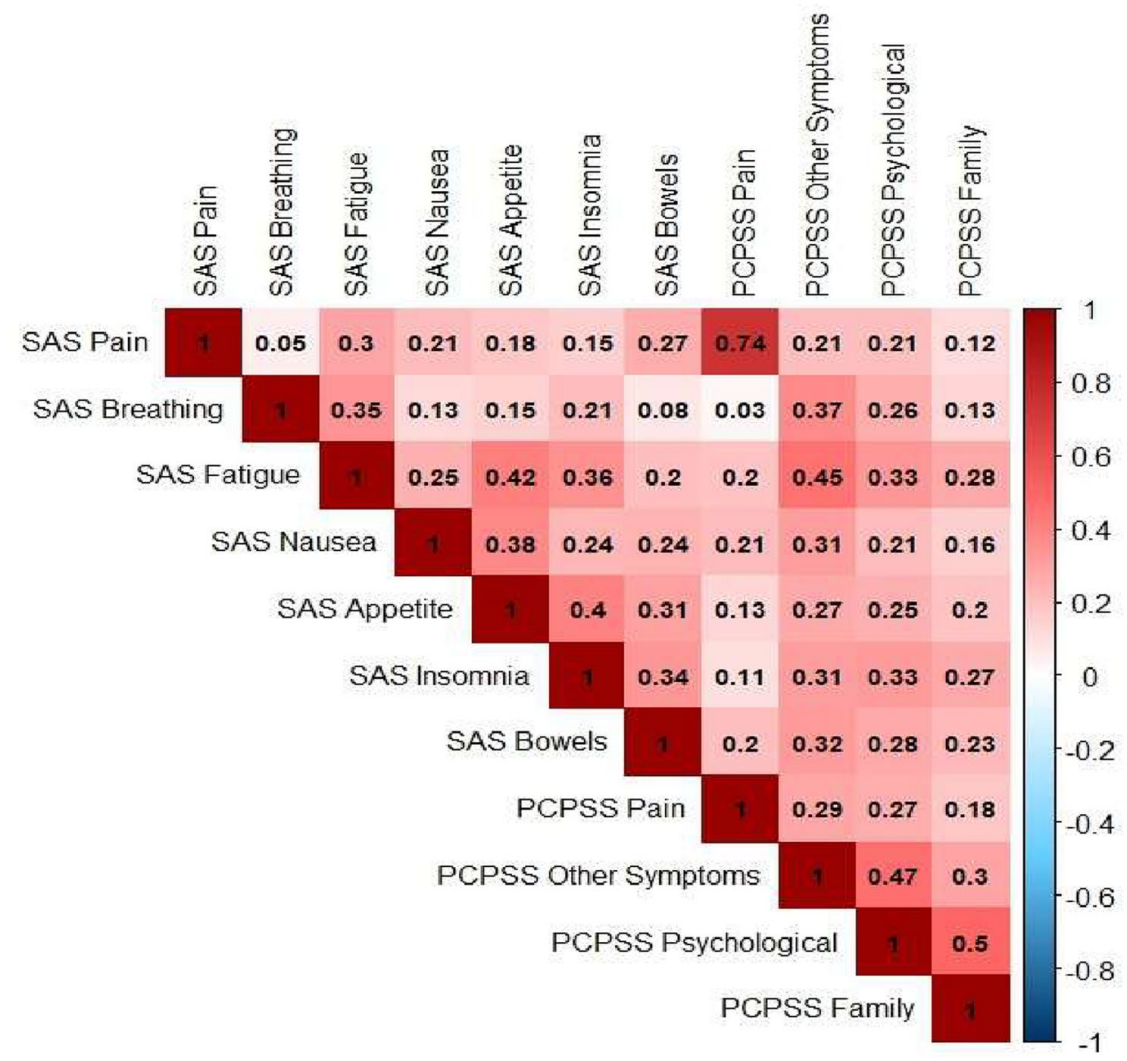
Convergent and discriminant validity between SAS and PCPSS items

Correlation analyses were conducted between related constructs to assess convergent validity between SAS and PCPSS. Accordingly, a strong correlation was observed between SAS pain and PCPSS pain, as hypothesised (*r* = 0.74). Discriminant validity was assessed by comparing unrelated constructs. The results showed weak correlations across most unrelated constructs of the two tools (Fig. 4).

### Known group comparison

As hypothesised, SAS demonstrated good discriminative ability in differentiating between stable and other palliative care phases in terms of pain (*p* = 0.004), nausea (*p* = 0.020), bowel problems (*p* = 0.007), poor appetite (*p* = 0.008). Additionally, it effectively differentiated symptom distress related to pain (*p* = 0.013) and breathing (*p* < 0.001) between cancer and non-cancer patients. Moreover, SAS effectively differentiated between inpatient and outpatient groups in terms of breathing (*p* = 0.011) and fatigue (*p* = 0.002).

The PCPSS effectively identified differences in pain (*p* < 0.001), psychological symptoms (*p* = 0.012), family/carer problems (*p* = 0.001), and other symptoms (*p* = 0.048) between phase types. In addition, significant differences in problem severity were observed between cancer and non-cancer patients in pain (*p* = 0.003), psychological symptoms (*p* = 0.024), and family/carer problems (*p* = 0.046). This tool also showed good discriminatory ability between inpatient and community settings for other symptoms (*p* = 0.049) and family/carer problems (*p* = 0.001).

The IPOS also demonstrated a strong ability to discriminate weakness for palliative care phases (*p* = 0.028). Moreover, the tool was able to differentiate symptoms between pain (*p* = 0.001), shortness of breath (*p* = 0.001), nausea (*p* = 0.043), and constipation (*p* = 0.001) between cancer and non-cancer patients. Finally, the tool significantly differentiated dry mouth (*p* = 0.048) and family anxiety (*p* = 0.032) (Table 5).

**Table 5:** Known group comparisons for SAS, PCPSS, and IPOS using the T-test or mann-whitney U test to assess construct validity

Tools	Symptoms	Phase type(Stable vs others)	Diagnosis(Cancer vs non-cancer)	Setting (Inpatient vs Community
SAS		**p-value**	**p-value**	**p-value**
	Pain	0.004^*^	0.013^*^	0.521
	Nausea	0.020^*^	0.207	0.080
	Insomnia	0.089	0.497	0.713
	Fatigue	0.237	0.075	0.002^*^
	Breathing	0.205	< 0.001^**^	0.009^*^
	Bowels	0.007^*^	0.669	0.830
	Appetite	0.008^*^	0.673	0.603
PCPSS	Pain	< 0.001^**^	0.003^*^	0.124
	Psychological	0.012^*^	0.024^*^	0.057
	Family/Carer	0.001^*^	0.046^*^	0.049^*^
	Other symptoms	0.048^*^	0.418	0.001^*^
IPOS Physical	Pain	0.690	0.001^**^	0.060
	Shortness of breath	0.174	0.011^*^	0.069
	Weakness	0.028^*^	0.054	0.706
	Nausea	0.281	0.043^*^	0.766
	Vomiting	0.345	0.143	0.073
	Poor appetite	0.109	0.279	0.419
	Constipation	0.493	0.001^**^	0.458
	Dry mouth	0.089	0.649	0.048^*^
	Drowsiness	0.133	0.147	0.262
	Poor mobility	0.109	0.761	0.552
IPOS Emotional	Patient anxiety	0.849	0.919	0.148
	Family anxiety	0.743	0.097	0.032^*^
	Patient depression	0.599	0.748	0.490
	Feeling at peace	0.180	0.406	0.327
IPOS information and practical issues	Sharing feelings	0.871	0.143	0.950
	Information	0.726	0.738	0.513
	Practical problems	0.198	0.870	0.936
IPOS	Total (t-test)	0.054	0.035^*^	0.400
IPOS	Physical (t-test)	0.009*	0.028^*^	0.584
IPOS	Emotional	0.435	0.384	0.311
IPOS	Communication and practical issues domain	0.843	0.456	0.796
SAS	Total	0.001^*^	0.444	0.814
PCPSS	Total	< 0.001^**^	0.536	0.092

Footnote: ^*^p < 0.05 (significant); ^**^p < 0.01 (highly significant): IPOS: Integrated palliative care Outcome Scale; SAS: Symptom Assessment Scale; PCPSS: Palliative Care Problem Severity Score

Patients whose episodes starting date before October 2020 (*n* = 53) were excluded from the IPOS known group comparisons to align with the survey collection period

## Discussion

The study found that the IPOS total has good reliability (α = 0.81), whereas the SAS and PCPSS demonstrated acceptable (α = 0.70) and marginal (α = 0.65) reliability, respectively. The emotional domain (four items) of the IPOS exhibited the highest reliability, followed by the physical domain (ten items), with lower reliability observed in the information and practical issues domain (three items). The study also found strong convergent validity for the pain constructs of the SAS and PCPSS measurements. The PCPSS and SAS showed overdominance in discriminating symptoms between palliative care phases and care settings in known-group comparisons. The physical domain of the IPOS has also demonstrated good discriminative ability in differentiating symptoms between cancer and non-cancer groups. Finally, a 3-factor model demonstrated strong structural validity for IPOS, verifying that the tool accurately reflects the physical, emotional, and practical domains.

Good internal consistency (α = 0.81) for the IPOS total in this study aligns with previous findings [11, 12, 20, 23]. However, the reliability for the information and practical issues domain was below the acceptance threshold. This low reliability for this specific domain is consistent with other previous studies and requires further exploration [11, 15, 20, 23]. Many possible factors might contribute to this outcome. The small number of items in this domain (only three) compared to other domains (physical: 10 items; emotional: 4 items) could affect Cronbach’s alpha, which is highly influenced by the number of items. In addition, weak inter-item correlations within the information and practical issues subdomain (0.11–0.26), as indicated by the inter-item correlation analysis, may also contribute to this result. Moreover, the components within this domain may capture heterogeneous aspects of practical skills that do not align well into a single construct. Alternatively, the items may give a limited or unbalanced view of the domain. Overall, the practical domain may require further conceptual refinement.

The IPOS is a multidimensional tool with three underlining domains: physical, emotional, and practical, as confirmed by our CFA. This is consistent with previous studies [15, 20]. Another study from Germany also identified the same three clusters (factors) for non-oncological patients [24]. However, the Czech Republic study found that a two-factor model best fitted their data; unfortunately, this study failed to conduct a CFA due to the limited sample size [10].

The reliability (α = 0.70) of SAS was acceptable and was slightly improved compared to previous Australian studies [2, 17] but still lower than that of a Chinese study [18]. The internal consistency of the PCPSS in this study was slightly lower than the acceptable range (α = 0.65), comparable with a previous Australian study [4]. Notably, the latter work used a different approach (Kappa coefficient) to assess PCPSS reliability and found moderate inter-rater agreement [4]. This finding is not surprising, as PCPSS has only four items, and internal consistency is highly influenced by the number of items. Another possible reason could be related to the inter-item correlations: the inter-item correlations for PCPSS (0.15–0.48) were slightly better than those for the SAS (0.08–0.43), but still weak. A further possible reason could be the way the two assessments were captured; the PCPSS was entirely clinician (proxy) rated, whereas the SAS could be rated by either clinicians or patients. Therefore, the small number of items, the weak inter-item correlations, and the proxy rating could all contribute to slightly lower reliability. However, stronger evidence, including a larger sample size and primary data, is warranted.

A strong correlation was detected between the pain constructs of SAS and PCPSS as hypothesised, indicating close alignment in pain assessment between these two tools, an observation previously noted [18]. The pain constructs of PCPSS and SAS are likely to be assessed by the same clinician, so a strong correlation is expected. On the other hand, there were weak correlations between most of the unrelated constructs of these two tools, suggesting good divergent validity. The convergent and discriminant validity between IPOS and the two tools was not discussed in this paper to reduce the introduction of bias because the data collection periods were different, as described in the method section.

The SAS effectively distinguished symptoms such as pain, nausea, bowel problems, and appetite problems between palliative care phases in known-group comparisons. Additionally, it identified significant differences in pain and breathing for cancer and non-cancer groups, as well as fatigue and breathing for community and inpatient settings. These findings are in line with previous studies [2, 18]. The PCPSS effectively discriminates between palliative care phases for all symptoms and for three symptoms (i.e., pain, psychological, family/carer problems, and other symptoms) between cancer and non-cancer groups. This tool also adequately differentiates family/carer problems and other symptoms between community and inpatient settings.

Another finding from the known group comparisons is that the mean score of the physical domain of IPOS is significantly higher for unstable/deteriorating/terminal phases compared to the stable phase and for cancer patients compared to non-cancer patients. Besides, the mean of the total IPOS is higher among cancer patients than non-cancer patients. Previous studies have also found that the IPOS discriminates the physical and emotional symptoms across phases and diagnoses [11, 25]. The IPOS effectivity differentiates between the inpatient and outpatient settings in two symptoms (i.e., dry mouth and family/carer anxiety). This finding aligns with a previous study [15]. The overdominance of the physical subdomain of IPOS is expected, as it is a part of a multidimensional domain tool.

The findings will assist palliative care programs, including PCOC, in identifying effective tools for comparing different groups and specific symptoms. They will also support cross-national comparisons, contributing to the advancement of benchmarking palliative care internationally. The results will further inform clinical assessment tool selection for outcome measurement, emphasising psychometric property robustness alongside comprehensiveness, as recommended by the 2016 White Paper on outcome measurement [1].

Furthermore, the PCOC program will utilise the findings to evaluate the measures used for quality improvement and benchmarking. We must consider the limitations and disparities between patient-reported and proxy-reported outcomes when selecting tools for symptom assessment. This consideration is particularly relevant, as patients receiving palliative care in community care settings are three times more likely to self-report when compared to those receiving palliative care in hospital settings [3]. This emphasises how important it is to carefully interpret PCPSS and SAS results and explore possible alternatives.

### Strengths and limitations

Some limitations should be considered when interpreting our findings. This study did not assess inter-rater agreement or test-retest reliability because it used routinely collected PCOC data to complement the original dataset. There was also considerable missing data (7.7%) for certain items, such as the practical issues item of IPOS. Lastly, slight variations in the data collection periods between the two datasets, the patient experience survey (collected from October 2020 to February 2021) and the PCOC longitudinal dataset (collected from July to December 2020), might have resulted in an underestimation or overestimation of symptoms. A prospective study with a large sample size and additional variables is recommended to address these limitations.

## Conclusion

In conclusion, tool selection for outcome measurement in palliative care programs, including PCOC, should be based on different considerations, such as their psychometric properties and the specific constructs they measure to ensure continued relevance as the field of palliative medicine evolves. It is also crucial to consider that outcome measures are suitable for national application in the Australian context, are adaptable to future changes, and can promote advances in palliative care through international benchmarking. Lastly, although IPOS, SAS, and PCPSS remain valid and reliable, they require further evaluations and modifications to address the future palliative care needs.

## Data Availability

All data are provided in the manuscript and accompanying supplementary files.

## Abbreviations

AKPS: Australia-modified Karnofsky Performance Status
CFI: Comparative Fit Index
CFA: Confirmatory factor analysis
IPOS: Integrated Palliative Care Outcome Scale
POS: Palliative Care Outcome Scale
PCOC: Palliative Care Outcomes Collaboration
PCPSS: Palliative Care Problem Severity Score
PROs: Patient-Reported Outcomes
RUG-ADL: Resource Utilisation Groups-Activities of Daily Living
RMSEA: Root-Mean-Squared Error of Approximation
SAS: Symptom Assessment Scale.
